# Application of VUV/Sulfite Defluorination System for the Simple Detection of Perfluoroalkyl Substances

**DOI:** 10.3390/molecules30112475

**Published:** 2025-06-05

**Authors:** Shiyong Tao, Yilin Chen, Xiao Mei, Luyao Jin, Feng Wu, Jing Xu

**Affiliations:** 1 State Key Laboratory of Water Resources Engineering and Management, Wuhan University, Wuhan 430072, China; taoshiyong@whu.edu.cn (S.T.); yilinchen0817@whu.edu.cn (Y.C.); meixiao@whu.edu.cn (X.M.); luyaojin@whu.edu.cn (L.J.); 2 Hubei Key Laboratory of Water System Science for Sponge City Construction, Wuhan University, Wuhan 430072, China; 3 Hubei Key Lab of Biomass Resource Chemistry and Environmental Biotechnology, School of Resources and Environmental Science, Wuhan University, Wuhan 430079, China

**Keywords:** PFAS, VUV/sulfite system, defluorination, hydrated electron, concentration analysis

## Abstract

This study investigated the defluorination of PFOA and PFOS using a vacuum ultraviolet (VUV)/sulfite system, and evaluated its potential application in quantifying individual perfluoroalkyl substances (PFAS). Results showed that 81.9% and 87.5% defluorination of PFOA and PFOS were achieved after 120 min of photoreaction under conditions of pH 12 and 20 mM of sulfite. Higher pH and sulfite dosage facilitated the reaction, while competing ions could suppress the defluorination efficiency. Based on the optimized defluorination conditions for individual PFAS, the potential of fluoride release amount, as an indirect quantification indicator, was further assessed. A strong linearity between the fluoride release and initial PFAS concentration (R^2^ > 0.999) was observed in the PFAS concentration range of 2–100 μM, and such linearity was also shown in the presence of sediment leachates. This correlation enabled the estimation of individual PFAS concentrations by measuring fluoride release after defluorination treatment. The approach was further demonstrated in an adsorption experiment, where calculated distribution coefficients (*K*_oc_) for PFAS–sediment interactions were consistent with previously reported values, supporting the analytical validity of the method under controlled conditions. Overall, this work presents a simple and cost-effective indirect analytical strategy of applying a VUV/sulfite defluorination system for individual PFAS quantitative detection in complex environmental matrices.

## 1. Introduction

Perfluoroalkyl substances (PFAS) are a class of synthetic organic pollutants with high environmental persistence and they have become a global research focus in environmental science, due to their ubiquitous distribution and ecological risks [1,2]. These chemicals exhibit excellent chemical stability and surfactant properties due to the multiple carbon–fluorine bonds (C-F), and are thus widely used in industrial manufacturing and consumer products [3,4]. During product usage and disposal, PFAS are inevitably released into the environment through multiple pathways, including wastewater discharge and landfill leachate leakage. PFAS have high water solubility due to the polar functional groups at their termini, facilitating cross-media migration through surface runoff and precipitation infiltration [5,6]. The combination of mobility and persistence leads to their widespread accumulation in aquatic systems, with detections even in glacial meltwater and atmospheric deposition [7,8]. In China’s Yangtze River Basin, many studies have reported the occurrence of PFAS in the major tributaries and adjacent lakes [9,10]. Among the various PFAS, perfluorooctanoic acid (PFOA) and perfluorooctane sulfonic acid (PFOS) are commonly used as representative model compounds due to their frequent detection in various environmental matrices and biota.

The analysis of PFAS involves a range of analytical techniques, each with specific advantages and limitations. Liquid chromatography–tandem mass spectrometry (LC-MS/MS) is regarded as the “gold standard” for its excellent sensitivity and selectivity, enabling trace-level quantification and structural identification of PFAS in complex matrices [4,11]. However, its high operational costs, specialized equipment, and the demand for well-trained personnel limit accessibility in resource-constrained institutions or regions [12]. Simpler techniques such as high-performance liquid chromatography (HPLC) with UV or fluorescence detection are more cost-effective but suffer from low sensitivity due to weak PFAS chromophores, and thus often require derivatization [13]. Total organic fluorine (TOF) analysis methods, such as combustion ion chromatography (CIC), photolysis-coupled ion chromatography analysis, fluorine-19 nuclear magnetic resonance (^19^F-NMR), and particle-induced gamma-ray emission (PIGE), have been developed to quantify total fluorine content [14,15,16,17]. While these TOF methods offer a view of total fluorine determination in the environmental medium, they still involve high costs or limited availability. Therefore, there is a demand to fill the gap between comprehensive but expensive methods and accessible routine analysis. These issues pose an important challenge in exploring simplified and cost-effective alternatives to facilitate PFAS analysis in the absence of advanced instruments.

Ultraviolet advanced reduction processes (UV-ARPs) are often considered for the reduction of compounds [18,19]. They utilize UV light to activate chemical reductants, and generate highly reactive species in water. In a representative UV/sulfite system, sulfite ions (SO_3_^2−^), which exhibit strong UV absorption, could generate hydrated electrons (e_aq_^−^) and sulfite radicals (SO_3_^•−^) via Equation (1) under the irradiation of UV light. The e_aq_^−^, with a reduction potential of −2.9 V, is recognized as one of the most powerful natural reductants and it can directly attack the highly electronegative fluorine atoms in PFAS molecules, destabilizing the C-F bonds and initiating sequential defluorination reactions [3,20]. Although e_aq_^−^ is quickly quenched by dissolved oxygen (*k* = 1.9 × 10^10^  M^−1^ s^−1^), its lifetime can be prolonged under inert gas conditions, thereby enhancing contaminant removal efficiency [21]. In addition, compared to conventional UV systems operating at 254 nm with a sulfite activation quantum yield of 0.39, vacuum ultraviolet (VUV) irradiation at 185 nm exhibits superior reactivity, achieving a higher quantum yield of 0.85 in sulfite-activated reduction systems [22]. Its higher photon energy enables the simultaneous activation of both sulfite and water molecules (Equation (2)), leading to the acceleration of pollutant degradation [23]. Experimental evidence has confirmed that VUV-driven advanced oxidation/reduction processes (AO/RPs) outperform conventional UV systems in eliminating UV-resistant micro-pollutants [24,25,26].

SO_3_^2−^ + *hν* → e_aq_^−^ + SO_3_^•−^ (1)

H_2_O + *hν* (<190 nm) → e_aq_^−^ + H^+^ + HO^•^ (2)

Given that fluoride release amount directly correlates with PFAS mineralization, the (V)UV/sulfite system holds potential not only to be used as a treatment approach, but also in conducting simple and economical quantification for individual PFAS through routine fluoride determination. Although UV-ARPs exhibit high treatment ability under ideal conditions, the ubiquitous presence of e_aq_^−^ quenchers in complex aqueous matrices can substantially compromise degradation efficiency, thereby affecting the defluorination extent. The common cations and anions in natural waters interact with reactive intermediates or modulate solution properties (e.g., pH, ionic strength, and light transmittance), thereby affecting the generation and reactivity of reactive species [24,27,28,29]. These ion-specific interactions may exert either inhibitory or synergistic effects on defluorination kinetics, depending on their chemical nature and concentration. Although the significance of ionic effects on the compound degradation has been widely recognized, understanding of their influences in VUV/sulfite-mediated defluorination remains limited. Investigating the influence of coexisting ions is also helpful for evaluating its applicability in the indirect determination of PFAS concentration.

In this study, the defluorination of two representative PFAS (PFOA and PFOS) was investigated using a VUV-activated sulfite system, with a focus on the influence of key operational parameters and environmentally relevant inorganic ions. The potential of released fluoride as an indirect quantification indicator was further assessed by evaluating its correlation with initial PFAS concentrations in both ultrapure water and sediment leachates. A simulated adsorption experiment was also conducted to demonstrate the practical applicability of this approach. This study provides a simple and cost-effective analysis process for laboratories lacking advanced instruments, enabling the quantification of individual PFAS in complex environmental matrices through fundamental laboratory procedures.

## 2. Results and Discussion

### 2.1. Defluorination of PFAS by VUV/Sulfite

#### 2.1.1. Effect of Irradiation Time on the Defluorination of PFAS

**Figure 1:**
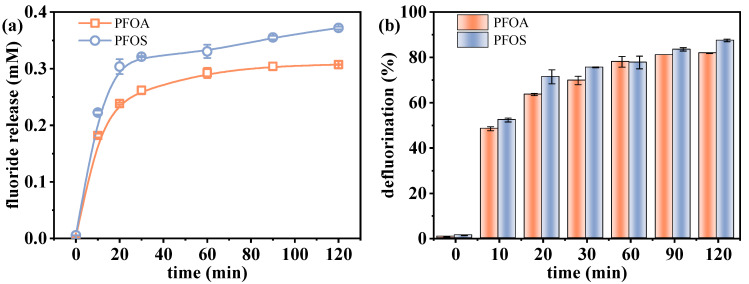
Changes in defluorination (**a**) amount and (**b**) efficiency with reaction time. Initial experimental conditions: [PFOA] = [PFOS] = 25 μM, [sulfite] = 20 mM, pH = 12.

The effect of VUV irradiation time on the defluorination performance of PFOA and PFOS was investigated. As shown in Figure 1, both compounds exhibited progressive fluoride releases, reaching 0.307 mM (81.9%) for PFOA and 0.372 mM (87.5%) for PFOS after 120 min. Although the defluorination efficiencies between PFOA and PFOS were quite similar, there existed slight differences in fluoride release amount, which were attributed to the different numbers of fluorine atoms in the parent molecules. Rapid defluorination occurred within the first 30 min, followed by a slower stage, likely due to sulfite depletion and the increasing stability of residual C–F bonds [30]. As the reaction proceeds, increased resistance to reduction occurs in residual C-F bonds, which are often situated at sterically hindered or electronically stable positions characterized by high bond dissociation energies [26,31]. Although UV-ARPs can achieve complete PFAS degradation with sufficient treatment time, defluorination is often incomplete [30,32]. For example, Bentel et al. [33] reported that while PFOA and PFOS were fully degraded after 8 h and 48 h UV treatment, respectively, their defluorination efficiencies were similar at ~50% over 48 h. This discrepancy arises because degradation can produce short-chain intermediates that retain fluorine, limiting the detectable fluoride release amount [34].

These results confirm the effectiveness of the VUV/sulfite system in facilitating the reductive defluorination of PFOA and PFOS, demonstrating its potential as a promising treatment approach for highly stable fluorinated pollutants. The fluoride release amount at 120 min was slightly higher than that at 60 min. Nevertheless, the time cost, whether for wastewater treatment or sample pretreatment, is an essential factor to consider. In this study, a reaction time of 60 min was selected for subsequent experiments to achieve a balance between defluorination efficiency and time cost.

#### 2.1.2. Effect of Initial pH on the Defluorination

**Figure 2:**
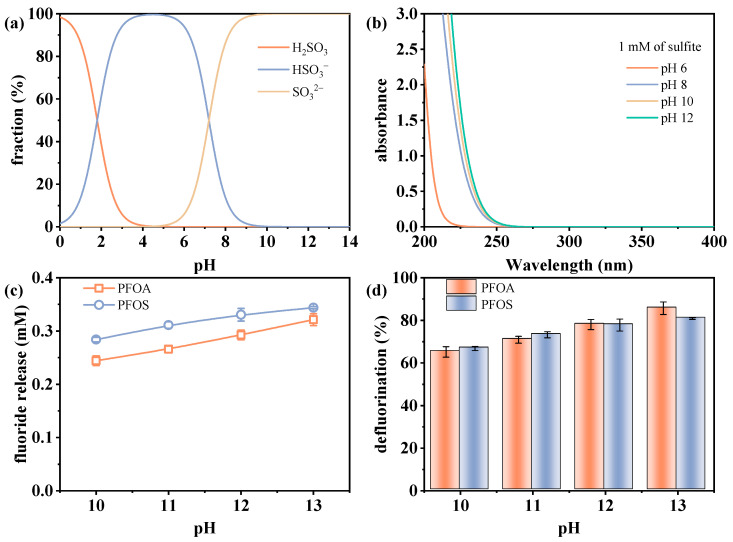
(**a**) Species distribution of sulfite, (**b**) absorbance of sulfite in UV range, and changes in defluorination (**c**) amount and (**d**) efficiency under different pH conditions. Initial experimental conditions in (**c**,**d**): [PFOA] = [PFOS] = 25 μM, [sulfite] = 20 mM, pH = 10–13, reaction time = 60 min.

The initial pH of the reaction solution plays a critical role in determining sulfite species, and subsequently influences the defluorination efficiency of perfluorinated compounds in the VUV/sulfite system. The two p*K*_a_ values of sulfite (1.8 and 7.2) [35,36] result in its predominant existence as a mixture of SO_3_^2−^ and HSO_3_^−^ in the pH range of 4–9, while SO_3_^2−^ becomes the dominant species at pH values above 9 (Figure 2a). The absorbance of sulfite increases under higher pH conditions, as shown in Figure 2b, making it more susceptible to photoexcitation under irradiation. In a previous work, Luo et al. [36] estimated that SO_3_^2−^ has a higher quantum yield than HSO_3_^−^ under photolysis at 266 nm (0.06 versus 0.01), with alkaline conditions conducive to enhancing the reaction efficiency.

Figure 2c,d present the defluorination performance of PFOA and PFOS under varying initial pH values from 10 to 13. The results show that the fluoride release amount of PFOA increased from 0.244 to 0.321 mM, and that of PFOS increased from 0.284 to 0.344 mM as the pH increased from 10 to 13, with corresponding defluorination efficiencies of 65.2–85.7% and 66.8–80.9%, respectively. The defluorination trends in Figure 2c,d confirm that alkaline conditions favor the photoreduction process in the UV/sulfite system. The observed trend is not only related to the pH-dependent photoactivity of sulfite but also to the propensity of e_aq_^−^ reacting with protons under acidic-to-neutral conditions (Equation (3)), which competitively reduces their availability for PFAS defluorination [37]. In contrast, under alkaline conditions, e_aq_^−^ is enhanced through reactions with H• and OH^−^ via Equation (4) [32], which increases degradation efficiency.

H^+^ + e_aq_^−^ → H^•^ (3)

H^•^ + OH^−^ → e_aq_^−^ + H_2_O (4)

#### 2.1.3. Effect of Sulfite Concentration on the Defluorination

**Figure 3:**
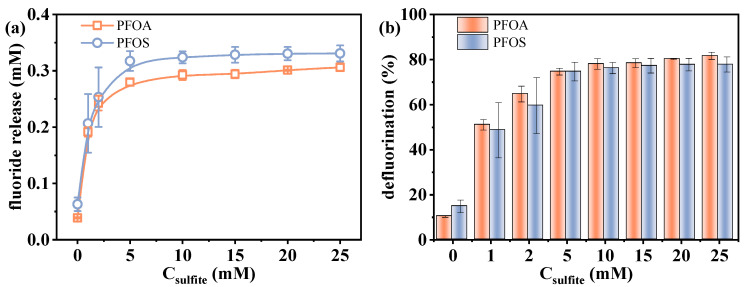
Changes in defluorination (**a**) amount and (**b**) efficiency with varied sulfite dosages. Initial experimental conditions: [PFOA] = [PFOS] = 25 μM, [sulfite] = 0–25 mM, pH = 12, reaction time = 60 min.

As a key source of e_aq_^−^, sulfite generates a higher concentration of e_aq_^−^ at elevated concentrations, thereby enhancing the reductive degradation process. The effect of varying sulfite concentrations (0–25 mM) on the defluorination performance was investigated. As shown in Figure 3, with the increase in sulfite concentration from 0 to 25 mM, the fluoride release amount of PFOA increased from 0.039 to 0.306 mM, and that of PFOS increased from 0.063 to 0.331 mM, corresponding to efficiencies of 10.3–81.7% and 14.8–77.9%, respectively.

Interestingly, in the absence of sulfite, over 10% defluorination was still observed for both PFOA and PFOS. This indicates that the VUV irradiation alone is capable of exciting water molecules to generate a limited amount of e_aq_^−^ (Equation (2)), which can also initiate partial PFAS degradation [26]. Direct photolysis may also make a certain contribution, as Xin et al. [38] found that the presence or absence of O_2_ had no significant influence on the photolysis rate of PFOA at 222 nm, suggesting a non-radical pathway. A sharp increase in defluorination was observed as the sulfite dosage increased from 0 to 5 mM. However, further increasing the sulfite concentration from 5 to 25 mM resulted in a slower rise in defluorination efficiency. Even at the highest sulfite concentration tested, complete defluorination was not achieved. As has been mentioned above, this deceleration is likely related to the increasing difficulty in cleaving the residual C-F bonds. In addition, although more sulfite generates more e_aq_^−^, excessive sulfite can also consume these electrons through side reactions (Equation (5)), limiting their availability for PFAS degradation [39]. Zhang et al. [25] also reported a two-stage trend (fast and slow) in the degradation rate constant of monochloroacetic acid in the VUV/sulfite system, demonstrating the side effect of the excessive sulfite. As a result, while sulfite is essential in enhancing defluorination, its overuse can counteract the system’s overall efficiency, emphasizing the necessity for optimal sulfite concentrations.

e_aq_^−^ + SO_3_^2−^ → Products (5)

### 2.2. Effect of Ions on the Defluorination of PFAS

In natural aquatic environments, PFAS coexist with a range of ions derived from natural sources or anthropogenic activities. These constituents can affect the physicochemical properties of the aquatic system, compete for reactive species, or interact directly with PFAS, and thereby alter the efficiency of degradation processes [40]. To better understand the VUV/sulfite system under realistic conditions, this section examines the effects of representative anions and cations abundant in surface waters on the defluorination efficiency of PFOA and PFOS.

#### 2.2.1. Effect of Anions

**Figure 4:**
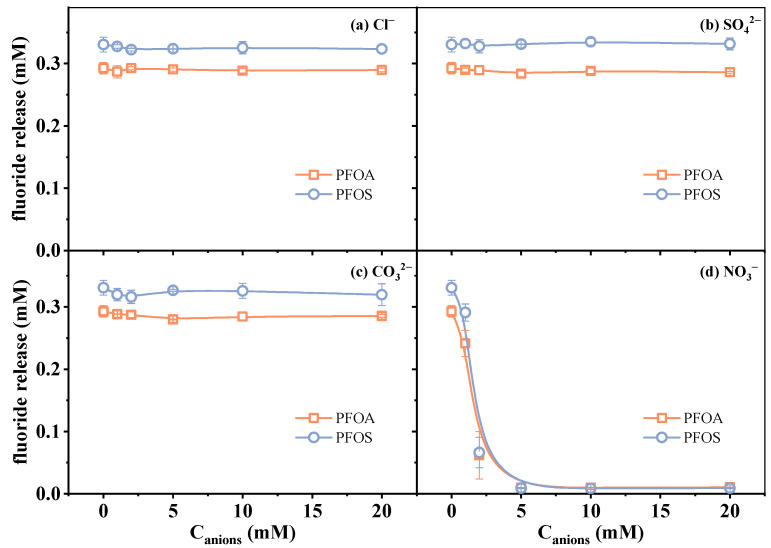
Changes in fluoride release amount in the presence of (**a**) Cl^−^, (**b**) SO_4_^2−^, (**c**) CO_3_^2−^, and (**d**) NO_3_^−^. Initial experimental conditions: [PFOA] = [PFOS] = 25 μM, [sulfite] = 20 mM, pH = 12, [Cl^−^] = [SO_4_^2−^] = [CO_3_^2−^] = [NO_3_^−^] = 0–20 mM, reaction time = 60 min.

The effects of four kinds of typical anions, including Cl^−^, SO_4_^2−^, CO_3_^2−^, and NO_3_^−^, on PFAS defluorination were investigated. Considering that coexisting ions may consume e_aq_^−^ and reduce defluorination efficiency, a 20 mM sulfite concentration was chosen in the following experiments to minimize these effects and provide a foundation for the quantification analysis using the VUV/sulfite system as a pretreatment approach. The experimental results of defluorination under varying ion concentrations (0–20 mM) are presented in Figure 4.

The results showed that Cl−, SO_4_^2−^, and CO_3_^2−^ exhibited a negligible influence on the fluoride release amount of both PFOA and PFOS under the investigated conditions. Previous studies on anions affecting the ARPs have reported distinct mechanistic pathways. It was reported that SO_4_^2−^ can be directly photolyzed into SO_4_^•−^ and e_aq_^−^, and a high concentration of SO_4_^2−^ could thus enhance the reductive reaction [24]. The defluorination efficiency in this work was not significantly improved, likely due to the lower dosage of SO_4_^2−^ compared to Liu et al.’s work [24]. Gu et al. [37] reported a slower reaction rate of PFOS when adding 1 mM of CO_3_^2−^ in the VUV/sulfite system at pH 10, which is considered to be caused by the competitive quenching of e_aq_^−^, though near-complete contaminant removal can still be achieved with extended reaction durations by compensating for scavenging effects. Such a negative effect on the reaction rate is more pronounced when using UV (254 nm) as a light source and increasing CO_3_^2−^ concentrations, as reported by Ren et al. [32]. In our work, the combination of elevated sulfite dosage (20 mM) and strongly alkaline conditions (pH 12) synergistically enhanced e_aq_^−^ yields, effectively masking the slight interference caused by CO_3_^2−^ which was reported under the conditions of lower reductant and lower pH value. The influence of Cl− on pollutant degradation varies with the reaction mechanisms, as it can either scavenge radicals or generate reactive chlorine species [41,42,43]. Under VUV irradiation, Cl− may also produce e_aq_^−^ (Φ_Cl−,185 nm_ = 0.43) or absorb light (ε_Cl−,185 nm_ = 3500 M^−1^ cm^−1^) [24], affecting reductive efficiency. However, in this study, its effect on defluorination was minimal under the tested conditions, possibly due to the combined influence of these factors. In contrast, the presence of NO_3_^−^ led to an obvious suppression of the defluorination efficiency. This inhibitory effect has been widely observed in many reported reductive systems [23,34,44]. Such an effect is attributed to the strong electron-scavenging ability of nitrate ions via Equation (6). This competitive reaction reduces the availability of e_aq_^−^ for attacking PFAS molecules, thereby hindering the reductive defluorination process. Moreover, the absorbance of NO_3_^−^ in the UV range would slightly decrease the photon flux available for sulfite photolysis, though this is likely a secondary effect [25,45].

NO_3_^−^ + e_aq_^−^ → (NO_3_)^•2−^ (6)

#### 2.2.2. Effect of Cations

**Figure 5:**
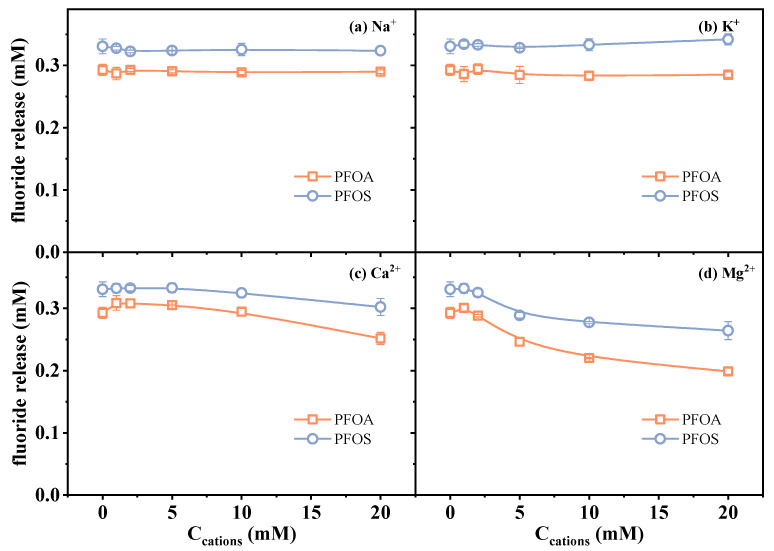
Changes in fluoride release amount in the presence of (**a**) Na^+^, (**b**) K^+^, (**c**) Ca^2+^, and (**d**) Mg^2+^. Initial experimental conditions: [PFOA] = [PFOS] = 25 μM, [sulfite] = 20 mM, pH = 12, [Na^+^] = [K^+^] = [Ca^2+^] = [Mg^2+^] = 0–20 mM, reaction time = 60 min.

To assess the potential effect of common cations in an aqueous environment, four kinds of cations, including Na^+^, K^+^, Ca^2+^, and Mg^2+^, were selected. Experimental results under varying ion concentrations (0–20 mM) are presented in Figure 5. The results showed that Na^+^ and K^+^ had negligible effects on the fluoride release amount of both PFOA and PFOS in the investigated concentration range. These two monovalent cations are commonly used as inert counterions in ionic strength influence studies, owing to their high solubility and minimal direct involvement in redox processes [46,47].

**Figure 6:**
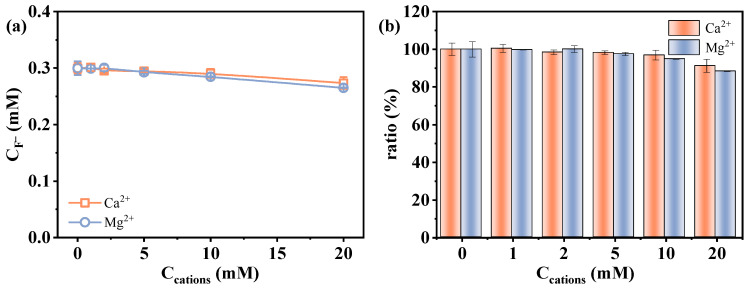
Effect of Ca^2+^ and Mg^2+^ on the determination of C_F^−^_ using fluoride ion-selective electrode: (**a**) detected F^−^ concentration and (**b**) F^−^ recovery. Conditions: [F^−^] = 0.3 mM, [Ca^2+^] = [Mg^2+^] = 0–20 mM.

Contrasting with monovalent cations, the presence of Ca^2+^ and Mg^2+^ led to a noticeable decrease in the measured fluoride release amount (Figure 5c,d). In order to distinguish whether it is the result of actual degradation inhibition or analytical deviation, fluoride recovery experiments were conducted by preparing standard fluoride solutions (0.3 mM F^−^) containing varied concentrations of Ca^2+^ or Mg^2+^ via an ion-selective electrode in the absence of PFAS and sulfite. The measured signal of F^−^ decreased progressively with increasing concentrations of Ca^2+^ and Mg^2+^ (91.2% and 88.4% at 20 mM Ca^2+^/Mg^2+^) (Figure 6). This phenomenon is likely attributed to the formation of less soluble fluoride salts. Fovet and Gal [48] demonstrated that Ca^2+^ and Mg^2+^ ions can significantly interfere with the potentiometric determination of F^−^ when using a fluoride ion-selective electrode. This interference is caused by the precipitation of fluoride salts (CaF_2_ and MgF_2_), which decreases the measurable fluoride ion activity. These results suggest that the observed decrease in defluorination may be related to the analytical deviation, i.e., precipitation leading to the decrease in detectable dissolved F^−^ concentration. This finding highlights the importance of considering ionic speciation and solubility when interpreting defluorination data in systems containing divalent metal ions. In addition, the influence of the two divalent metal cations on reductive reaction efficiency may also be related to factors such as PFAS–cation complex formation [5], as the decrease in defluorination was observed to exceed the signal loss induced by the precipitation of fluoride salts (Figure 5c,d vs. Figure 6).

Overall, these results demonstrate that although monovalent cations have a minimal effect, divalent cations such as Ca^2+^ and Mg^2+^ can interfere with the determined amount of defluorination due to the reasons of both the analytical technique and reduction efficiency. This underscores the necessity of incorporating ion-matrix correction strategies into studies of PFAS degradation.

### 2.3. Application of VUV/Sulfite System in PFAS Quantification Analysis

#### 2.3.1. Establishment of Calibration Curves

**Figure 7:**
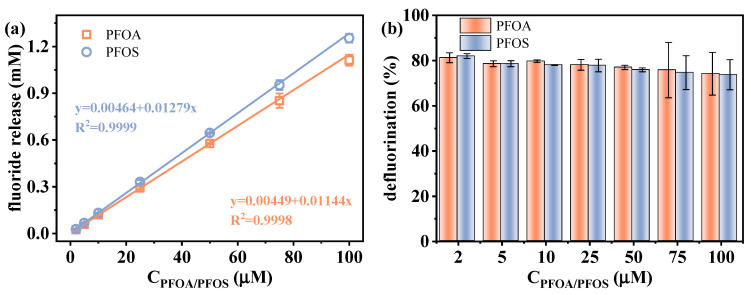
(**a**) Relationship between the fluoride release amount and initial PFAS concentrations, and (**b**) defluorination efficiency of PFOA/PFOS under varied initial PFAS concentrations. Initial experimental conditions: [PFOA] = [PFOS] = 2–100 μM, [sulfite] = 20 mM, pH = 12, reaction time = 60 min.

The feasibility of applying the VUV/sulfite system for the indirect quantification of individual PFAS via defluorination is investigated. The defluorination performance of PFOA and PFOS was examined under varying initial concentrations (2–100 μM). As shown in Figure 7, a strong linear correlation (R^2^ > 0.999) was observed between the fluoride release amount and initial PFAS concentrations, though there was a slight decrease in defluorination efficiency with increasing PFAS concentrations due to the fixed generation rate of e_aq_^−^ under constant VUV/sulfite conditions. This indicates that, under sufficient sulfite dosage, the system is capable of achieving a stable response to various PFAS concentrations up to 100 μM. Such excellent linearity provides a solid foundation for indirect PFAS determination. The limit of detection (LOD) was calculated according to Equation (7):

LOD = 3 × SD(7)

where SD represents the standard deviation of blank sample measurements. The obtained LODs for these two PFAS were 0.0549 and 0.0492 μM, respectively. In addition, the measurement accuracy was evaluated by repeatedly determining the sample with a PFAS concentration of 25 μM 10 times. As shown in Table 1, the calculated recoveries of PFOA and PFOS were 100.8% and 101.9%, and the relative standard deviations (RSDs) were 2.95% and 3.63%, respectively. These results reflect the robustness of the defluorination efficiency of the VUV/sulfite system in ultrapure water medium, which enables PFAS quantification by determining the fluoride release amount after reductive pretreatment.

**Figure 8:**
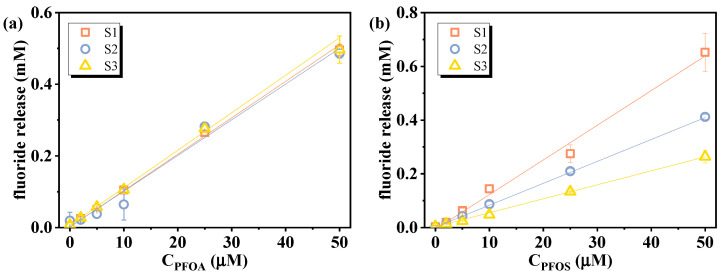
Calibration curves of (**a**) PFOA and (**b**) PFOS under the influence of sediment leachates. Initial experimental conditions: [PFOA] = [PFOS] = 2–50 μM, [sulfite] = 20 mM, pH = 12, reaction time = 60 min.

Then, the effect of sediments on the correlation between the fluoride release amount and initial PFAS concentrations was assessed using leachates from three sediments (S1, S2, S3) spiked with 2–50 µM of PFOA or PFOS and treated with VUV/sulfite. As shown in Figure 8 and Table 2, both PFOA and PFOS retained excellent linearity, despite an overall decrease in defluorination efficiency relative to ultrapure water. This decrease may be attributed to NO_3_^−^ and divalent metal ions dissolved from the sediments, as has been discussed above. Additionally, sediment-derived dissolved organic matter (DOM) can quench e_aq_^−^ and reduce VUV light utilization efficiency through competitive photon absorption [25,49]. PFOA calibration curves remained similar across all sediments, whereas PFOS showed greater variation, likely due to its stronger complexation tendency, which may reduce its availability for e_aq_^−^-driven reduction [5,50]. To counteract these effects and improve the response signal, optimization of operational parameters (such as sulfite dosage and treatment duration) is necessary. Using actual environmental matrices to establish calibration curves not only counteracts the matrix-induced effect of defluorination efficiency but also helps eliminate the influence of background fluoride in the sample, since both standards and test samples undergo identical processing. For samples with high PFAS concentrations, dilution can reduce matrix effects while maintaining detectability. Mild pretreatment (e.g., UV pre-irradiation or solid-phase extraction to remove DOM) may also be considered prior to defluorination analysis to reduce their impact.

Although this method was demonstrated using PFOA and PFOS, it is conceptually applicable to a broader range of PFAS. Previous studies have shown that e_aq_^−^-based systems have the ability to degrade various organofluorines, including short-chain PFAS, perfluoro-carboxylates and perfluoro-sulfonate, though defluorination efficiency may vary with molecular structure, particularly for ultra-short-chain or more stable compounds [24,30,33]. Similar to TOF-based approaches, this method measures total fluoride release amount and cannot distinguish the contributions of each PFAS in a mixture, which limits its applicability for mixed PFAS analysis. Nevertheless, these results demonstrate that, under appropriate conditions, fluoride release remains a reliable indirect measurement for individual PFAS quantification in complex medium.

#### 2.3.2. Application in Adsorption Experiment

**Figure 9:**
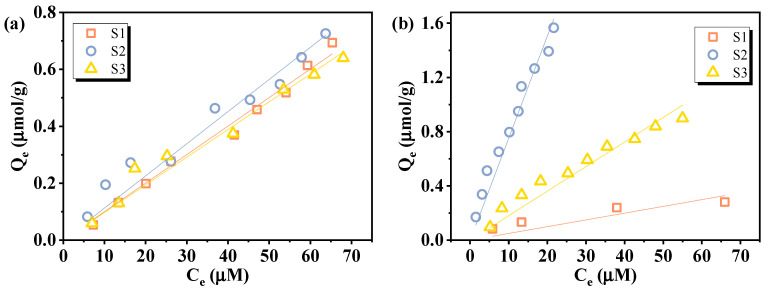
Adsorption isotherms of (**a**) PFOA and (**b**) PFOS onto sediments.

Based on the established calibration curves in Figure 8, the practical applicability of the VUV/sulfite defluorination system in PFAS quantification was explored through adsorption experiments. The equilibrium concentration of PFAS in the aqueous phase was determined by applying the VUV/sulfite system. Then, the adsorption isotherms of PFOA and PFOS onto three different sediment samples were evaluated, as shown in Figure 9. Both compounds exhibited measurable adsorption to all three sediments, confirming their affinity for natural solid phases. PFOA exhibited similar adsorption behaviors onto the three sediments, while PFOS showed more obvious variability, which may be attributed to its higher susceptibility to the constitution of sediments due to its longer chain [51].

Linear fitting was applied to the sorption isotherm data to calculate the partitioning coefficient (*K*_d_, L/kg) and organic carbon-normalized distribution coefficient (*K*_oc_) according to Equations (8) and (9):

*K*_d_ = Q_e_/C_e_ (8)

*K*_oc_ = *K*_d_/*f*_oc_ ×100 (9)

where Q_e_ (μmol/g) is the concentration of PFAS in the sediment, C_e_ (μmol/mL) is the equilibrium concentration of PFAS in water, and *f*_oc_ is the fraction of organic carbon in the sediment (given in Table 3), respectively. *K*_oc_ enables a standardized comparison of sorption capacities across sediments with varying organic carbon contents. The calculated log*K*_oc_ values for PFOA ranged from 2.25 to 2.29, and those for PFOS ranged from 1.98 to 3.07, both within the range of values previously reported in the literature [51,52,53]. These results validate the feasibility of employing the VUV/sulfite system as a quantitative tool for analyzing perfluorinated compounds in complex environmental matrices, which offers a promising approach for assessing PFAS behavior in natural sorption processes.

## 3. Materials and Methods

### 3.1. Chemicals and Sediments

PFOA (96%) and PFOS (98%) were purchased from Macklin Biochemical (Shanghai, China). Sodium sulfite (Na_2_SO_3_), sodium hydroxide (NaOH) and other reagents were purchased from Sinopharm Chemical Reagent Co., Ltd., Shanghai, China. Ultrapure water with 18.2 MΩ cm resistivity, used in all experiments, was obtained from a water purification system (Ming-Che 24UV, Millipore, France). The sediments used in this work were sampled from the river channels in Wuhan City, China. The collected sediment samples (S1, S2 and S3) were dried at room temperature and protected from light. Then, the dried solids were ground and sieved using a 100-mesh sieve.

### 3.2. Photochemical Reaction

**Figure 10:**
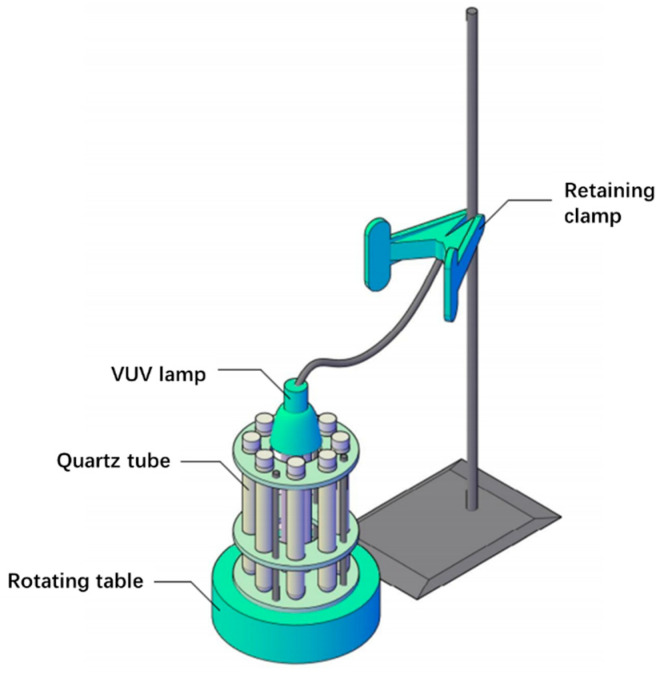
Photoreactor structure diagram.

The structure of the reactor used in this work is shown in Figure 10. A VUV disinfection lamp containing light at 185 nm was used as the irradiation light source. A total of 8 quartz tubes were used as the reaction tubes, as quartz allows the penetration of the VUV light. A circular reaction rack was placed on the rotating table, and the VUV lamp was placed in the center of the reaction rack, with 8 quartz tubes evenly placed around the VUV lamp. In the light reaction process, the rotating table was turned on so that the quartz tubes rotated around the ultraviolet lamp to avoid reaction differences due to the light intensity. Each quartz tube was equipped with a PTFE plug to isolate the circulation of air inside and outside of the reaction tube.

In order to investigate defluorination efficiency of PFOA/PFOS in the VUV/sulfite system under different conditions, solutions containing the desired amount of several reagents were prepared, including PFOA/PFOS as the reaction substrate, NaOH which provided the alkaline conditions, and anions or cations when needed. After each reagent was added into the quartz tube, ultrapure water was added to make up the total reaction volume to 10 mL. Then, a certain amount of sodium sulfite powder was added into the tube. The mixed reaction solution was aerated under argon (99.999%) for 30 min to remove the dissolved oxygen. After aeration, the quartz tube was quickly plugged with a PTFE plug to isolate the solution from contact with air. The prepared quartz tubes were then placed on the reaction rack, and the VUV lamp was turned on to start the reaction. When the desired reaction time was reached, we turned off the VUV lamp to stop the reaction. All experiments were conducted at least in duplicate.

### 3.3. Simulated Adsorption Experiments

Batch adsorption experiments were conducted in 15 mL centrifuge tubes under controlled conditions (25 °C) for 24 h. A weight of 0.5 g of sediment was equilibrated with 10 mL of PFAS solution under varied initial concentrations. After 24 h of adsorption, 5 mL of the filtered reaction solution was transferred into a quartz tube and subjected to photoreaction treatment according to the procedure described in Section 3.2. The fluoride concentration in the solution was then measured after the treatment using the VUV/sulfite system. In order to establish a fluoride–PFAS calibration curve under the influence of the sediment leachates, ultrapure water was used to prepare a control mixture with the same solid-to-liquid ratio as that in the adsorption experiment. This mixture was shaken for 24 h to extract soluble components from the sediment. The extract was then transferred into a quartz tube, spiked with a known concentration of PFAS, and subjected to the same photoreaction process as described in Section 3.2.

### 3.4. Analytical Methods

The released fluoride ion (F^−^) was measured by an ion-selective electrode (PXSJ-216F, Shanghai Yidian Analysis Instrument Co., Ltd., Shanghai, China) according to the Chinese standard of GB 7484-87 [54] (water quality–determination of fluoride–ion-selective electrode method). TISAB III (total ionic strength adjustment buffer, containing (CH_2_)_6_N_4_, KNO_3_, and C_6_H_4_Na_2_S_2_·H_2_O) was used during the analysis to avoid further pH adjustment. The property of sediments was analyzed according to the national standard methods. The spectrum of sulfite under varied pH conditions was recorded using a UV-Vis spectrophotometer (UV-3600, Shimadzu, Japan) using a 1 cm quartz cuvette at room temperature.

## 4. Conclusions

In this study, the defluorination of PFOA and PFOS using a VUV/sulfite system was investigated, with an exploration of its potential application in PFAS quantification. High defluorination efficiency of both PFOA and PFOS was achieved within 60 min of reductive reaction under the conditions of 20 mM of sulfite and pH 12. Operational parameters, including irradiation time, initial pH, and sulfite concentration, affected the defluorination efficiency: a higher pH value, higher dosage of sulfite and longer reaction time all enhanced the defluorination efficiency. The effects of common environmental ions occurring in surface waters were also evaluated. NO_3_^−^ and divalent cations (Ca^2+^ and Mg^2+^) showed inhibitory effects, likely due to electron scavenging and precipitation, while other ions such as Cl−, SO_4_^2−^, CO_3_^2−^, Na^+^, and K^+^ exhibited negligible effects under the experimental conditions. Calibration curves established under varying PFAS concentrations exhibited excellent linearity (R^2^ > 0.999), and maintained robustness even in the presence of sediment leachates, despite a reduction in absolute fluoride release amount. Furthermore, the VUV/sulfite defluorination system was successfully applied to quantify the PFAS concentration in a sediment adsorption experiment. Both PFOA and PFOS showed measurable adsorption behaviors, and the calculated log*K*_oc_ values were consistent with those reported in the literature. Overall, this work highlights the dual function of the VUV/sulfite system, offering an effective approach for PFAS degradation, and a rapid, cost-effective and reliable indirect analytical strategy for quantifying individual PFAS species in complex environmental matrices.

## Figures and Tables

**Table 1:** Detection limit and measurement accuracy of PFOA and PFOS.

Compounds	Blank (μM)	Measurement ± SD (μM)	LOD of PFAS (μM)	PFAS (μM)	Recovery (%)	RSD (%)
PFOA	0	0.0185 ± 0.0183 0.0048 ± 0.0164	0.0549	25	100.8	2.95%
PFOS			0.0492		101.9	3.63%

**Table 2:** Equations of calibration curves under the influence of sediment leachates.

Sediments	Compounds	Equation	R^2^
S1	PFOA	y = 0.01017x + 0.0012	0.9988
	PFOS	y = 0.01290x − 0.0064	0.9649
S2	PFOA	y = 0.00997x + 0.0018	0.9941
	PFOS	y = 0.00816x + 0.0015	0.9986
S3	PFOA	y = 0.01047x + 0.0062	0.9974
	PFOS	y = 0.00520x + 0.0030	0.9980

**Table 3:** Calculated water–sediment partitioning parameters for PFOA and PFOS.

Sediments	*f*_oc_ (%)	PFOA			PFOS		
		log*K*_d_	log*K*_oc_	R^2^	log*K*_d_	log*K*_oc_	R^2^
S1	5.18	1.00	2.29	0.9966	0.70	1.98	0.9241
S2	6.35	1.05	2.25	0.9898	1.88	3.07	0.9898
S3	5.08	0.99	2.28	0.9919	1.26	2.55	0.9878

## Data Availability

The data that support the findings of this study are available from the authors upon reasonable request.
